# Effects of Interleukin-6 Dysregulation in a Mouse Model of Alzheimer’s Disease: Unraveling Complexity Beyond Amyloidosis

**DOI:** 10.3390/cells15211921

**Published:** 2026-10-22

**Authors:** Carla Canal, Kevin Aguilar, Gemma Comes, Amalia Molinero, Mercedes Giralt, Paula Sanchis, Juan Hidalgo

**Affiliations:** 1 Animal Physiology Unit, Department of Cell Biology, Physiology and Immunology, Faculty of Biosciences, Universitat Autònoma de Barcelona, Bellaterra, 08193 Barcelona, Spain; carla.canal@uab.cat (C.C.); kevin.aguilar@irbbarcelona.org (K.A.); gemma.comes@uab.cat (G.C.); am.molinero55@gmail.com (A.M.); merce.giralt@uab.cat (M.G.); 2 Institut de Neurociències, Universitat Autònoma de Barcelona, Bellaterra, 08193 Barcelona, Spain; 3 Institute for Research in Biomedicine (IRB Barcelona), 08028 Barcelona, Spain; 4 Centre for Physical Activity Research (CFAS), Rigshospitalet, University of Copenhagen, 2100 Copenhagen, Denmark

**Keywords:** Alzheimer’s disease, Tg2576 mice, Interleukin-6, neuroinflammation, amyloid-β, APP

## Abstract

**Highlights:**

**What are the main findings?**

• In this specific cohort, aged Tg2576 mice displayed physiological, behavioral, and cognitive alterations despite the absence of detectable cerebral amyloid-β and amyloid plaques.
• Interleukin-6 deficiency modulated several Tg2576-associated phenotypes, with more pronounced effects in females.

**What are the implications of the main findings?**

• Amyloid precursor protein overexpression-associated phenotypes can emerge independently of high Aβ40 and Aβ42 levels and amyloid plaque deposition.
• Interleukin-6 contributes to the physiological and behavioral phenotype of Tg2576 mice in a sex- and age-dependent manner.

**Abstract:**

Interleukin-6 (IL-6) is a pleiotropic cytokine implicated in neuroinflammation and Alzheimer’s disease (AD) pathophysiology, but its contribution to the phenotype associated with amyloid precursor protein (APP) overexpression remains unclear. Here, we investigated the effects of systemic IL-6 deficiency in Tg2576 mice by assessing survival, physiological parameters, behavior, learning and memory, APP/amyloid-β (Aβ)-related pathology, and gliosis in young and aged male and female mice. In this specific cohort of animals, Tg2576 mice exhibited reduced survival and body weight, together with age- and sex-dependent alterations in behavioral and cognitive parameters. Notably, despite increased circulating Aβ40 and Aβ42, Aβ40 and Aβ42 were undetectable in the brain, and amyloid plaques were absent in Tg2576 mice compared with plaque-positive control mice. IL-6 deficiency partially rescued several of these phenotypes, particularly in females. Thus, the APP overexpression-associated phenotype observed here occurred in the absence of detectable cerebral amyloidosis, highlighting that APP overexpression-associated alterations can manifest independently of amyloid plaque deposition. Together, our findings underscore the complexity of AD pathogenesis and emphasize the multifaceted nature of IL-6 in both physiological and neurodegenerative processes, particularly in the context of AD.

## 1. Introduction

Alzheimer’s disease (AD) is one of the most common neurodegenerative diseases and the primary cause of dementia in the elderly [1]. It is characterized by initial short-term memory loss followed by progressive cognitive decline. The two major neuropathological hallmarks of AD are extracellular amyloid deposits of amyloid-β peptides (Aβ) and intracellular neurofibrillary tangles of hyperphosphorylated tau protein, together with extensive neuronal dysfunction, synaptic loss, and vascular damage in the affected brain regions, such as hippocampus and cortex [2,3]. Aβ peptides are generated through sequential cleavage of the amyloid precursor protein (APP) by β-secretase 1 and the γ-secretase complex [4], and their accumulation and aggregation have traditionally been considered central drivers of AD pathogenesis [5]. Aβ pathology is also closely associated with neuroinflammation, characterized by the activation of astrocytes and microglia and the release of inflammatory mediators that can contribute to disease progression and severity [6,7].

Several disease-associated mutations in *APP*, presenilin 1 (*PSEN1*), and presenilin 2 (*PSEN2*) have been identified in familial forms of AD [8]. A substantial gain of knowledge on the mechanisms underlying amyloid pathology in AD has been achieved through the development and analysis of transgenic mouse models overexpressing human APP (*hAPP*) carrying disease-promoting mutations associated with early-onset AD in humans [9]. However, these models differ considerably in the onset and spatial distribution of Aβ pathology, neuronal loss, and learning and memory deficits, limiting the extrapolation of general conclusions regarding the pathogenic contributions of Aβ peptides [10]. Detailed characterization of the transgene expression pattern and the resulting pathology is therefore essential for understanding the mechanisms underlying the phenotype of each model. The Tg2576 mouse, established by Hsiao and colleagues [11], is one of the most widely used transgenic models and overexpresses *hAPP* carrying the Swedish double mutation KM670/671NL. Tg2576 mice develop Aβ deposits beginning in the entorhinal cortex and subsequently extending to the hippocampus at approximately 11 months of age [11,12,13] and display age-associated behavioral and cognitive alterations, including increased activity and exploratory behavior, reduced anxiety-like behavior, impaired spatial learning, and deficits in working memory, together with Aβ oligomer formation and extracellular amyloid deposition [11,14,15,16,17].

Importantly, however, accumulating evidence indicates that APP overexpression can produce functional and pathological alterations that are not necessarily dependent on amyloid plaque deposition. In particular, intracellular APP C-terminal fragments not cleaved by γ-secretase, including C99, have been implicated in early APP-related pathological processes and cognitive alterations [18,19,20,21,22]. In mice, the formation of soluble Aβ and C-terminal APP fragments requires transgenic expression of *hAPP* [23]. However, differences in *hAPP* expression across neuronal populations and brain regions may contribute to specific pathological and cognitive disturbances observed in Tg2576 mice before amyloid plaque pathology becomes apparent [23]. Altered APP signaling has also been associated with dysregulation of synaptic dynamics and downstream cellular pathways independently of Aβ toxicity [24]. Together, these findings suggest that the Tg2576 phenotype may reflect a combination of amyloid-dependent and amyloid-independent mechanisms and that APP overexpression should not necessarily be equated with cerebral amyloidosis.

Neuroinflammation represents a potential mechanism linking APP dysregulation to the functional and pathological alterations observed in AD models. Interleukin-6 (IL-6) is a pleiotropic multifunctional cytokine, with both pro- and anti-inflammatory actions, which participates in numerous physiological processes [25]. In the central nervous system, IL-6 has been implicated in the regulation of behavioral and cognitive functions, suggesting that it may have an impact on neurodegenerative diseases [26,27]. Increased IL-6 expression has been reported in serum, brain tissue, especially in the area surrounding senile plaques, and cerebrospinal fluid of AD patients [28,29], as well as in Tg2576 mice, in which IL-6 has been detected around and within β-amyloid plaques [30]. In addition, IL-6 and other pro-inflammatory cytokines (such as Interleukin-1β and Tumor necrosis factor-α) can promote APP production and up-regulate β-secretase 1 activity, potentially enhancing Aβ production and establishing a positive feedback loop [28,31]. Conversely, IL-6 may also exert protective effects by enhancing gliosis and promoting Aβ clearance through phagocytosis [32,33]. Furthermore, studies in IL-6-deficient mice indicate that IL-6 contributes to the regulation of locomotor and exploratory activity, anxiety-like behavior, and memory under physiological conditions [34,35,36]. Thus, the effects of IL-6 in AD may be context-dependent and influenced by disease stage, cellular source, and the underlying pathological environment.

Previous work has shown that modulation of IL-6 trans-signaling, one of the major signaling pathways with significant pro-inflammatory actions, as well as chronic IL-6 transgenic expression in astrocytes, can influence behavioral and physiological phenotypes in Tg2576 mice [17,37], but the contribution of systemic IL-6 to the broader phenotype associated with *hAPP* overexpression remains unclear. Here, we investigated the effects of systemic IL-6 deficiency on survival, physiological parameters, behavior, learning, and memory in young and aged male and female Tg2576 mice. We further examined APP-/Aβ-related pathology and gliosis to determine whether the effects of IL-6 were associated with cerebral amyloid pathology. Our findings show that IL-6 has a significant impact on the behavioral phenotype of the AD mouse model even in the absence of significant increases in Aβ levels, suggesting a potential role for IL-6 in the development of the disease.

## 2. Materials and Methods

### 2.1. Animals

The parental strains used in this study were the Tg2576 mice expressing the human *APP* harboring the Swedish K670N/M671L mutations (B6;SJL mixed background, purchased from Taconic Europe for this experiment) [11] and the IL-6-DIO-KO mice recently generated in our laboratory (C57BL/6 background backcrossed for more than ten generations) [38]. Heterozygous Tg2576 (APP_SWE_^+/−^) mice were mated with homozygous IL-6-DIO-KO (IL-6^−/−^) mice following a two-step breeding strategy to obtain the desired offspring: control (APP_SWE_^−/−^/IL-6^+/−^), referred to as wild-type (WT) in the text, IL-6KO (APP_SWE_^−/−^/IL-6^−/−^), Tg2576 (APP_SWE_^+/−^/IL-6^+/−^) and Tg2576/IL-6KO (APP_SWE_^+/−^/IL-6^−/−^). Genotype was determined by PCR analysis of tail DNA as previously described [11,38]. In selected experiments, several Tg2576 mice exhibiting well-established amyloid-β plaque pathology from other founders were used as positive controls (C+) to rule out methodological problems.

The experimental unit is a single animal, and the four groups were compared with each other. Sample size was based on previous experience with this mouse model of AD. Because of its known mortality and the variability of behavioral studies (the primary outcome measures), large groups start the experiments. The number of mice per group is indicated in the Figure legends. The total number of animals was 419. Offspring of the breeding pairs were randomly distributed between the different groups in several rounds (2–8 per breeding pair). The different genotypes were mixed in their cages, randomly, separated by sex, and as littermates (2–6 per cage depending on the number of pups and sex). No criteria for excluding animals were set. We avoided confounding factors such as the order of measurements by randomly mixing the genotypes, sexes and cages. The experimenters were not aware of the identity of the animals while carrying out the behavioral tests.

Mice were group-housed with littermates in a temperature-controlled environment (22 ± 2 °C) under specific pathogen-free conditions, with a 12 h dark-light cycle and were maintained with *ad libitum* food and water. Environmental enrichment was set (tissue paper in the cages). Experiments were conducted during the light phase, and mice were monitored regularly from weaning (P24–28) to euthanasia at 18 months of age, with both male and female mice used, unless otherwise noted. The general aspect of the animals (piloerection, abnormal postures), motor activity and reactive behavior to handling and body weight loss (0–3 points for each) were controlled weekly to identify distress and putative need of further supervision by experienced veterinary personnel. Umbral points were established for humane endpoints (>6 points; or 3 points in each category if persistent for >48 h).

### 2.2. Behavioral Tests

Mice were tested at ~5–6 and at ~16–17 months of age to characterize their behavioral phenotype as described previously [17,37,39]. Locomotor activity, exploratory activity, and anxiety were evaluated with the Open Field (OF), Hole Board (HB), and Elevated Plus Maze (EPM) tests, respectively; spatial memory and spatial learning were assessed with the Morris water maze (MWM), and working memory was assessed with the Y-Maze (YM). A week of handling adaptation was provided to the mice before testing and each test was preceded by a 30 min acclimation period in the home cage in the testing room. Tests were conducted in the previously specified order on alternate days.

#### 2.2.1. OF, HB, and EPM Tests

The OF test was used to evaluate locomotor and exploratory activity by measuring ambulatory distance and rearing (considered when the animal rises on its hindlimbs and extends its head upwards). The apparatus consisted of a rectangular white methacrylate box (36.5 × 56 × 31 cm) and mice were placed in a corner and allowed to freely explore the apparatus for 5 min.

The HB test was used to assess exploratory behavior by measuring time and number of head-dipping events (considered when the head of the animal was introduced to the holes at least to its ear level). Mice were placed in a square white methacrylate box (40 × 40 × 21.5 cm) with four equally spaced bottom holes (3 cm diameter) and were allowed to freely explore the apparatus for 5 min.

The EPM test was used to assess anxiety-like behavior by determining the percentage of entries and time spent in the open arms (OAs) of the apparatus (percentage from entries or time to both open and closed arms, excluding the center). Mice were placed in the center of a cross-shaped white methacrylate maze consisting of two open arms (20 × 5 cm) and two closed arms (20 × 5 × 15 cm) connected by a central square (5 × 5 cm) and elevated 47 cm from the floor and were allowed to freely explore the apparatus for 5 min.

All tests were recorded by an overhead video camera, and videos were analyzed with the Noldus Ethovision program version 11 (Wageningen, the Netherlands). Data were obtained through both in situ manual measurements (rearings and head-dipping events) and automatic tracking analysis (total distance moved and time and entries to the OA).

#### 2.2.2. MWM and YM Tests

The MWM apparatus consisted of a round pool (120 cm diameter × 60 cm deep) virtually divided into four equal quadrants with a removable platform (8 cm diameter). The pool was filled with opaque water (containing white tempera) at 22 ± 1 °C and surrounded by cues (4 2D cues on the walls (sticked black geometrical shapes: square, cross, triangle and circle) and 4 3D cues in the surrounding curtain (red ball, green plastic box, blue plastic cups and white palette)).

The test aimed to assess learning and hippocampal-dependent memory, and animals were tested for 11 days in differentiated phases, as described in the following protocol [17]. On day 1, animals underwent a 60 s probe trial with no platform or distal cues to assess potential visual or swimming deficits, followed by a four-trial cued-learning session (60 s per trial) with a visible platform to assess potential quadrant preferences but no distal cues. During cued learning, the platform location and starting position were changed between trials.

The platform was fixed in the southwest (SW) quadrant, whereas the starting positions varied across trials (north [N], east [E], southeast [SE], and northwest [NW]). Each trial lasted up to 60 s; if an animal failed to locate the platform within this time, it was guided to the platform and required to remain there for 15 s. Short-term memory was assessed in a probe trial 1 h after the last training session, and long-term memory was assessed in a second probe trial 24 h later (day 6). For both probe trials, the platform was removed, and mice were allowed to swim freely for 30 s.

From days 2 to 5 (spatial acquisition), animals underwent four trials per day to locate a hidden platform submerged 1 cm below the water surface in a target quadrant, using distal cues as spatial references. The platform remained fixed in the southwest quadrant, while the starting position varied across trials (north, east, southeast, and northwest). Trials were separated by an inter-trial interval of approximately 1 h and lasted up to 60 s. If an animal failed to locate the platform within this time, it was guided to the platform and allowed to remain there for 15 s. Short—(1 h after the last training session) and long-term (24 h after, on day 6) memories were tested in the probe trials, in which the platform was removed, and mice were allowed to freely swim for 30 s. During the spatial reversal learning and memory (days 7 to 11), animals were challenged to locate the platform at a new position in the northeast quadrant, using the same procedure as during spatial acquisition. The starting positions varied across trials (south, west, northwest, and southeast). Each trial was recorded by an overhead video camera and analyzed using the ANY-maze version 5.1 (Stoelting Europe, Dublin, Ireland). Escape latency (time to find the hidden platform) was recorded for each trial in the spatial acquisition and reversal phases, and the mean of the four trials was represented for each day of training. The area under the curve (AUC) was calculated for each escape latency curve. Latency to reach the area where the platform (platform area; PA) was located (±5 cm) and time in the target quadrant (TQ) were measured in the probe trials.

In the YM test [40], mice were placed at the end of the starting arm of a white methacrylate Y-shaped maze consisting of three closed arms (33 × 5 × 15 cm) oriented at 120º from each other and with external cues placed around the apparatus. A single trial was performed, in which mice were allowed to freely explore the apparatus for 8 min. Arm entries (considered when all four limbs of the mouse were within an arm) were manually recorded, and alternations (considered as consecutive entries into all three arms) were calculated to determine the percentage of alternation behavior as number of alternations/(total number of arm entries − 2) * 100.

#### 2.2.3. Nesting Behavior

During the testing period, mice were individually housed in a cage with bedding on the floor. To facilitate nesting behavior, pre-weighed, soft cotton nesting material (3 g of Nestlet) was provided one hour before the onset of the dark cycle. Nests were assessed the following morning (one hour after the light cycle started). Nesting scores were calculated by the extent of shredded material and the shape of the nest constructed on a scale of 1–5 (from undisturbed material to near perfect nest with complete shredding and construction of a full dome-like nest) according to published criteria [41]. Only female mice were included in the study, and photos were taken for scoring purposes.

### 2.3. Tissue Preparation

Mice were weighed and euthanized by decapitation at 18 months of age. Liver and fat depots, including brown adipose tissue (BAT) located on the back, and subcutaneous inguinal white adipose tissue (WAT), were dissected and weighed. BAT, WAT and liver relative weights were calculated as absolute weight/total body weight × 100. The tibia was dissected and measured with a caliper. Brains were extracted and the left hemisphere was fixed in 4% paraformaldehyde (PFA) for 24 h, stored in 70% ethanol at 4 °C, and processed for paraffin embedding and subsequent histology. The right hemisphere was dissected into cortex and hippocampus, frozen in liquid nitrogen, and stored at −80 °C. Then, samples were homogenized by sonication (Labsonic^®^ P, Sartorius, Ontario, Canada) in a lysis buffer containing 50 mM Tris-HCl pH 7.6, 150 mM NaCl, 2 mM EDTA, 0.1% Igepal CA 630 (Fluka, Sigma-Aldrich Chemie GmBH, Steinheim, Germany), 3% SDS, and 2% sodium deoxycholate, supplemented with a 1:100 protease inhibitor cocktail and 1 mM phenylmethylsulfonyl fluoride (Sigma-Aldrich, St. Louis, MO, USA). Total tissue protein quantification was performed with the Pierce^TM^ BCA Protein Assay Kit (Thermo Scientific, Rockford, IL, USA) according to the manufacturer’s protocol and absorbance was measured at 570 nm (Victor3 1420 Multilabel Counter, Perkin Elmer, Waltham, MA, USA). Whole blood was collected, and the resulting serum was separated by centrifuging at 8000 rpm for 10 min at 4 °C to separate the resulting supernatant as serum. Both the total homogenate and serum samples were stored at −80 °C for analysis.

### 2.4. Neuropathological Analysis

#### 2.4.1. Western Blotting for Amyloid Cascade

Western blot (WB) for APP, Aβ and other APP-derived proteolytic fragments was carried out as previously described [17,37]. Briefly, proteins were separated on a NuPage^TM^ 4–12% Bis-Tris Midi protein Gel (Invitrogen, ThermoFisher Scientific, Bender MedSystems GmBH, Vienna, Austria) by electrophoresis and transferred to 0.2 mm nitrocellulose membranes. Membranes were blocked with 5% (*w*/*v*) skim milk and then incubated with primary antibodies (mouse anti-β-amyloid_1–16_ 6E10 1:2000, 803003 Biolegend, San Diego, CA, USA; rabbit anti-GFAP 1:40,000, Z0334 Dako, Glostrup, Denmark; and anti-Iba-1 1:3000, 019–19741 Wako, Osaka, Japan) overnight at 4 °C. Secondary antibodies (polyclonal rabbit anti-mouse Ig/HRP 1:5000, P0260 Dako, Glostrup, Denmark; goat anti-rabbit IgG (H + L)-HRP conjugate 1:5000 and 1:10,000, 1706515 Bio-Rad Laboratories, Hercules, CA, USA) were then applied, and immunoreactive bands were detected using the Clarity Western ECL Substrate (Bio-Rad Laboratories, Hercules, CA, USA), imaged in the Chemidoc Imaging System (Bio-Rad Laboratories, Hercules, CA, USA), and analyzed using Image Lab^TM^ 6.0 Software (Bio-Rad Laboratories, Hercules, CA, USA). Monoclonal anti-β-actin (1:5000, Sigma-Aldrich, St. Louis, MO, USA) was used as a loading control following membrane stripping with 2% SDS, Tris HCl, and Tris Base pH 6.7.

#### 2.4.2. Enzyme-Linked ImmunoSorbent Assay (ELISA)

Amyloid beta 40 and 42 Human ELISA Kits were used to measure Aβ_1–40_ and Aβ_1–42_ (KHB3481 and KHB3341, Invitrogen, ThermoFisher Scientific, Bender MedSystems GmBH, Vienna, Austria) in cortex, hippocampus and serum samples. The protocol was performed according to the manufacturer’s recommendations.

#### 2.4.3. Immunohistochemistry (IHC)

Paraffin-embedded brain samples were cut into 8 mm sagittal sections to assess Aβ plaque load and gliosis in cortex and hippocampus. Briefly, slides were deparaffinized, treated with 70% methanol and 2% H_2_O_2_ for peroxidase inhibition, and then blocked with 0.5% *w*/*v* bovine serum albumin (BSA) in TBS-Triton 0.5%. A 20 min pre-treatment with 70% formic acid was necessary for the detection of Aβ plaques. Slides were then incubated overnight at 4 °C with primary antibodies against Aβ (mouse anti-β-amyloid_17–24_ 4G8 1:5000, Biolegend, San Diego, CA, USA), GFAP (rabbit anti-GFAP 1:900, Dako, Glostrup, Denmark), and IBA-1 (rabbit anti-IBA-1, 1:1500, Wako, Osaka, Japan) in 0.1% (*w*/*v*) BSA in TBS-Triton 0.5%. Subsequently, slides were incubated with the biotinylated secondary antibodies against mouse IgG (goat anti-mouse IgG (Fc specific) 1:400, Sigma-Aldrich, St. Louis, MO, USA) and rabbit IgG (goat anti-rabbit IgG (H+L) 1:300, ATOM, Manchester, UK), and then with Horseradish Streptavidin Peroxidase (Vector Laboratories, Newark, NJ, USA). 3,3′ Diaminobenzidine (DAB) was used as the chromogen. Finally, slides were dehydrated and mounted with DPX mounting medium.

For quantification, two non-consecutive sections per mouse were selected and imaged using a Nikon Eclipse 90i microscope (Nikon Corporation, Tokyo, Japan). Images were analyzed using ImageJ software 1.53t, and for both cortex and hippocampus, the area occupied by specific signal was measured.

### 2.5. Statistics

Data are shown as mean ± SEM and plotted using GraphPad Prism version 9. Statistical analysis was conducted using SPSS version 23. Separate analyses were performed for male and female samples. Survival was evaluated using the Kaplan–Meier and Log-rank survival tests, with genotype as a four-level factor (WT, IL-6KO, Tg2576, and Tg2576/IL-6KO). The rest of the data were analyzed either with a Generalized Linear Model (GzLM) with hAPP and IL6-KO as main factors (e.g., number of rearings, time in the OA) or Generalized Estimated Equations (GEEs) for repeated measures, including both genotypes as main factors and time as a within-subject factor (e.g., escape latencies in the MWM). The GzLM tolerates different distributions, heterogeneity of variances, and missing values and tests for the two main effects and the interaction between them. Statistical significance was set as *p* ≤ 0.05. Sample size is stated in the text or the figure as needed.

### 2.6. Availability of Aata

The raw data supporting the conclusions of this article will be made available by the authors on request.

## 3. Results

### 3.1. IL-6 Deficiency Partially Rescues Mortality in Female Tg2576 Mice Without Reversing Tg2576-Associated Weight Loss

**Figure 1:**
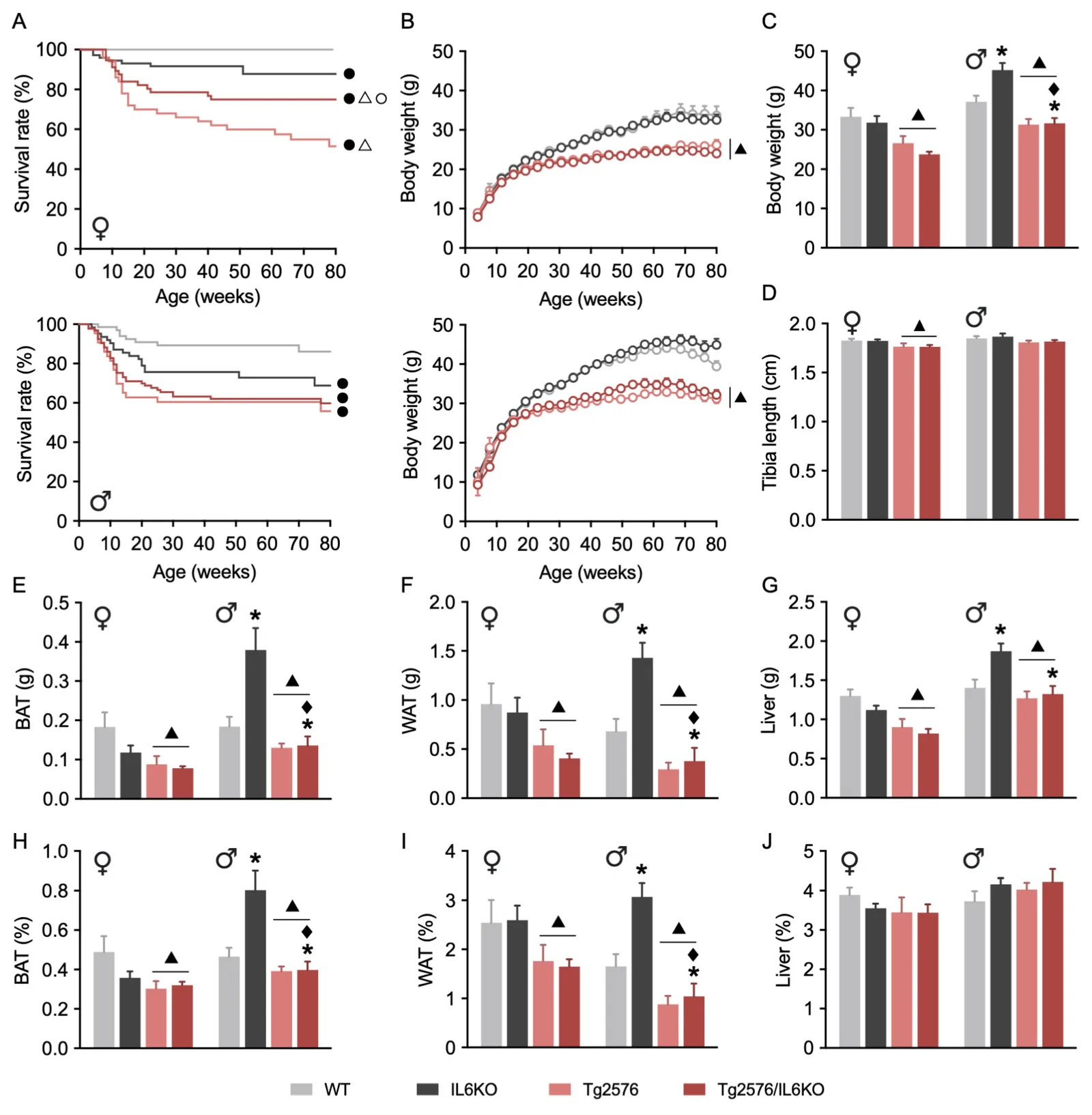
Effect of IL-6 deficiency on lifespan, body weight, and physiological variables in Tg2576 mice. (**A**) Kaplan–Meier survival curves showing decreased survival in Tg2576 mice, which was partially rescued by IL-6 deficiency in females. (**B**) Body weight was decreased in Tg2576 mice compared with their littermate controls; IL-6 deficiency did not modify this effect but increased body weight in males per se. (**C**) Body weight at 18 months was decreased in Tg2576 mice and increased in IL-6-deficient males. (**D**) Tibia length was decreased in female Tg2576 mice, with no significant effect of IL-6 deficiency. (**E**–**G**) Absolute weights of brown adipose tissue (BAT) (**E**), white adipose tissue (WAT) (**F**), and liver (**G**) followed similar trends. (**H**–**J**) Relative weights of brown adipose tissue (BAT) (**H**), white adipose tissue (WAT) (**I**), and liver (**J**) followed similar trends. Data are presented as mean ± SEM. ● *p* ≤ 0.05 vs. WT; △ *p* ≤ 0.05 vs. IL-6KO; ○ *p* ≤ 0.05 vs. Tg2576; ▲ *p* ≤ 0.05 Tg2576 effect; * *p* ≤ 0.05 IL-6 deficiency effect; ♦︎ *p* ≤ 0.05 interaction between both factors. n = 20–33 females and n = 13–26 males at 80 weeks of age for survival and body weight analyses.

A significant decrease in survival occurred in both male and female Tg2576 mice, with approximately a 45% reduction observed at 80 weeks of age. IL-6 deficiency partially rescued the mortality observed in female Tg2576 mice, but not in males. IL-6 deficiency also reduced survival in both male and female IL-6KO mice, although to a lesser extent (Figure 1A).

Tg2576 animals also showed decreased body weight compared to littermate control mice in both sexes throughout their lifetime, but IL-6 deficiency had no impact on this trait (Figure 1B). Interestingly, IL-6-deficient males developed mature-onset obesity compared to WT males, as evidenced by increased body weight and fat depots upon euthanasia. No differences regarding body weight and fat depots were observed in IL-6-deficient females compared to WT females. Absolute weights of BAT and WAT and, to a lower extent, liver, were decreased in Tg2576 mice in both sexes, and no major changes were observed with concomitant IL-6 deficiency. Similar results were observed for fat-relative weights, but not for the liver, which did not change significantly (Figure 1C,E–J).

Additionally, tibia length was found to be significantly lower in female Tg2576 females, with only a tendency towards a decrease in males, with no effect from IL-6 deficiency (Figure 1D).

### 3.2. Activity, Exploration, and Anxiety-like Behavior of Tg2576 Mice Are Modulated by IL-6 Deficiency

**Figure 2:**
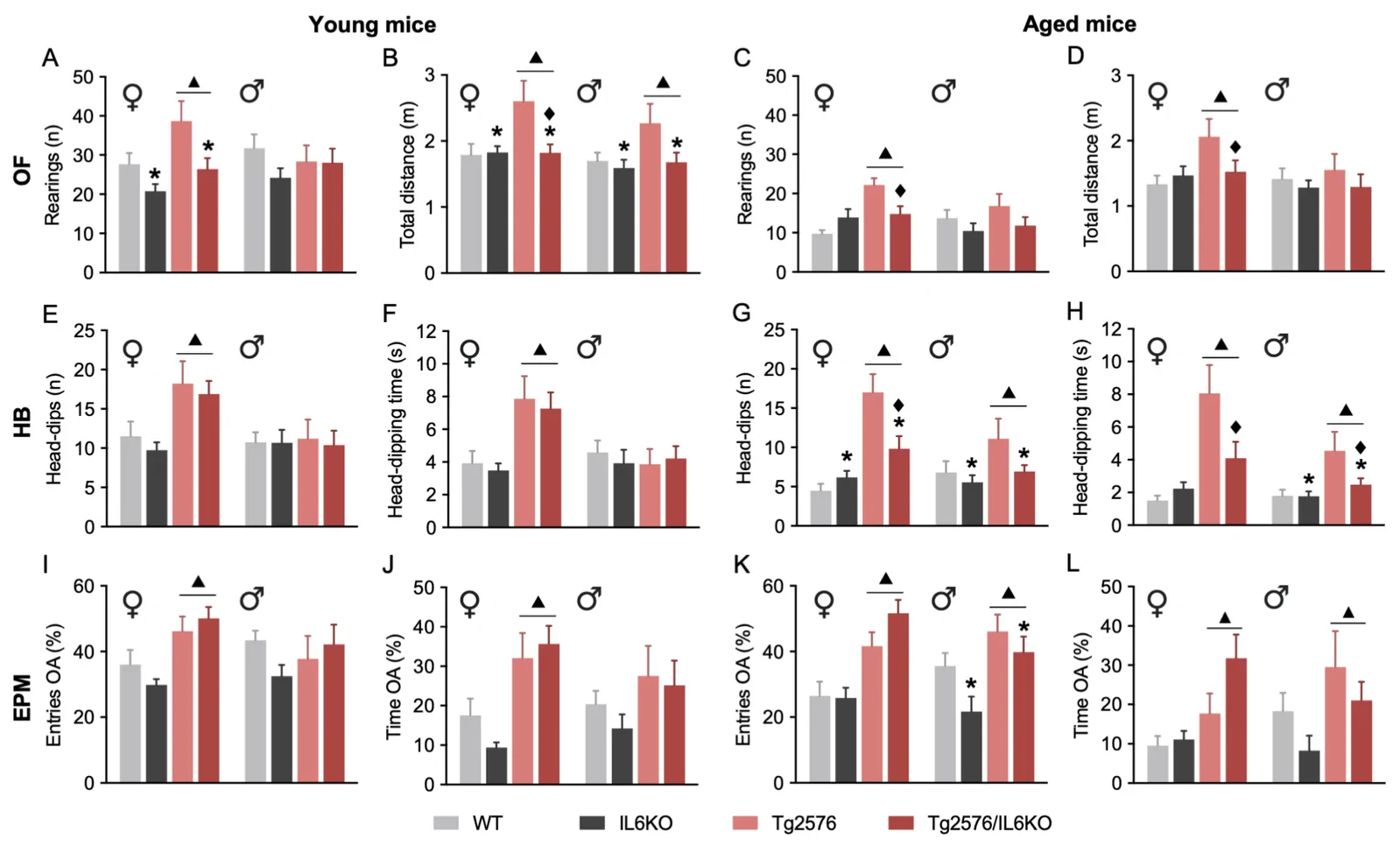
Effect of IL-6 deficiency on behavior in young and aged Tg2576 mice. (**A**,**B**) Increased vertical (**A**) and horizontal (**B**) activity in young Tg2576 females was partially attenuated by IL-6 deficiency. (**E**,**F**) Increased exploration and (**I**,**J**) decreased anxiety-like behavior in young Tg2576 females was not rescued by IL-6 deficiency. (**C**,**D**) Increased activity in aged females, rescued by IL-6 deficiency. (**G**,**H**) Increased exploration and (**K**,**L**) decreased anxiety-like behavior in old Tg2576 mice, partially rescued by IL-6 deficiency. Results are mean ± SE; n = 16–27 in females, n = 12–19 in males (**A**,**B**,**E**,**F**,**I**,**J**); n = 11–17 in females, n = 10–16 in males (**C**,**D**,**G**,**H**,**K**,**L**). ▲ *p* ≤ 0.05 Tg2576 effect, * *p* ≤ 0.05 IL-6 deficiency effect, ♦︎ *p* ≤ 0.05 interaction between both factors.

The effect of IL-6 deficiency on behavioral traits previously observed in Tg2576 mice was tested in both young (5–6 months old) and aged (16–17 months old) mice. As expected, young Tg2576 females showed clear signs of hyperactivity in the OF (increased ambulation and rearings; horizontal and vertical activity, respectively), increased exploration in the HB (increased time and number of head-dipping events), and decreased anxiety in the EPM (increased percentage of entries and time spent in the open arms of the maze), changes mostly present in females (Figure 2A,B,E,F,I,J). Young Tg2576 males only showed an increase in ambulation. IL-6 deficiency attenuated the increased activity of Tg2576 females and males but did not modify their exploratory and anxiety phenotypes; no clear-cut effects were observed compared to WT mice.

In old Tg2576 mice, similar behavioral phenotypes were generally observed in both males and females. IL-6 deficiency again reversed the hyperactivity, but also the increased exploratory behavior; moreover, it tended to reverse the decreased anxiety in male mice but to potentiate it in female mice (Figure 2C,D,G,H,K,L). As in young mice, no clear-cut effects of IL-6 deficiency were observed compared to WT mice, except for increased anxiety in male mice.

### 3.3. IL-6 Influences Learning and Memory Impairment of Tg2576 Mice

We next sought to evaluate the effect of IL-6 deficiency on hippocampal-dependent tasks such as spatial memory and learning in the MWM paradigm and spatial working memory in the YM paradigm.

**Figure 3:**
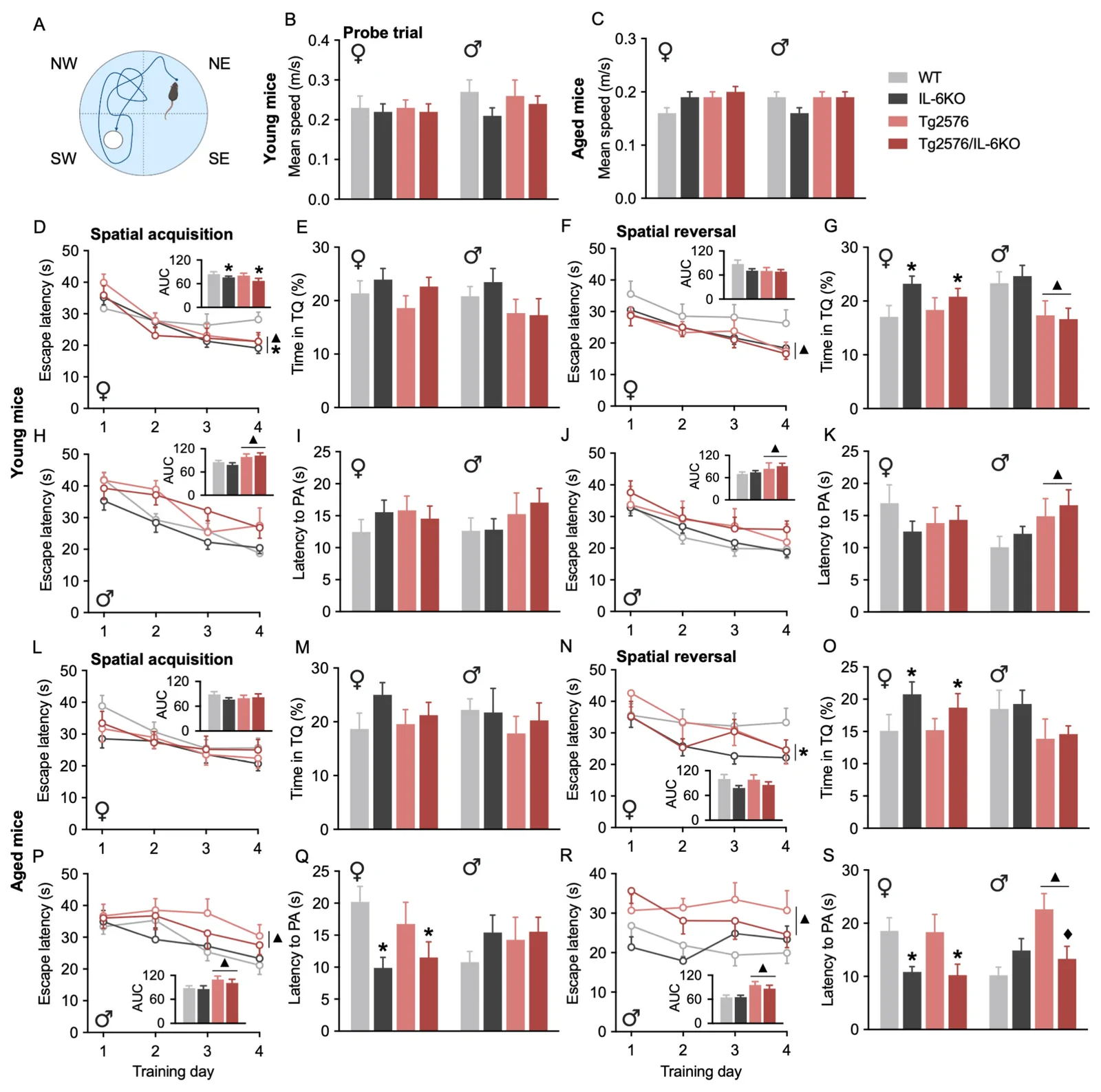
Effect of IL-6 deficiency on learning and memory in young and aged Tg2576 mice. (**A**) Schematic representation of the MWM apparatus. (**B**,**C**) No significant effects on mean swimming speed during the probe trial in young and aged mice. (**D**,**E**,**H**,**I**) Impaired spatial acquisition in young Tg2576 mice, and improved acquisition in IL-6-deficient females. (**F**,**G**,**J**,**K**) Impaired spatial reversal in young Tg2576 mice and retention in Tg2576 males, improved retention in IL-6-deficient females. (**L**,**M**,**P**,**Q**) Impaired spatial acquisition in aged Tg2576 males, improved retention in IL-6-deficient females. (**N**,**O**,**R**,**S**) Impaired spatial reversal and retention in aged Tg2576 males and improved in IL-6-deficient females. Results are mean ± SEM; n = 14–27 in females, n = 10–20 in males (**A**–**H**); n = 9–16 in females, n = 10–14 in males (**I**–**P**). ▲ *p* ≤ 0.05 Tg2576 effect, * *p* ≤ 0.05 IL-6 deficiency effect, ♦︎ *p* ≤ 0.05 interaction between both factors.

In the Morris water maze, no significant effects on mean swimming speed during the probe trial were observed in young and aged mice (Figure 3A–C). Young Tg2576 male but not female mice showed impaired learning, evidenced by increased escape latency and AUC in the spatial acquisition phase. In contrast, IL-6 deficiency improved learning in females, but not male mice, as seen by a reduction in escape latency and AUC, in both Tg2576 and WT backgrounds, thus suggesting a role of IL-6 in learning in young females. No changes were observed in long-term memory consolidation in young mice, as indicated by a comparable time in the target quadrant and latency to the platform area (Figure 3D,E,H,I). In the spatial reversal test, memory consolidation was impaired in Tg2576 male mice and improved in IL-6-deficient female mice at both ages (Figure 3F,G,J,K).

In aged mice, learning deficits persisted in Tg2576 males, even though no effects were found in long-term memory. In contrast, aged Tg2576 females did not display any learning or memory deficits (Figure 3L,M,P,Q). However, IL-6-deficient females showed improved learning and long-term memory, demonstrated by a reduction in escape latency, as well as increased time in TQ and decreased latency to PA in both spatial acquisition and reversal phases. In males, IL-6 deficiency improved long-term memory in an APP-dependent manner, as demonstrated by a decrease in latency to PA in the spatial reversal phase (Figure 3N,O,R,S).

**Figure 4:**
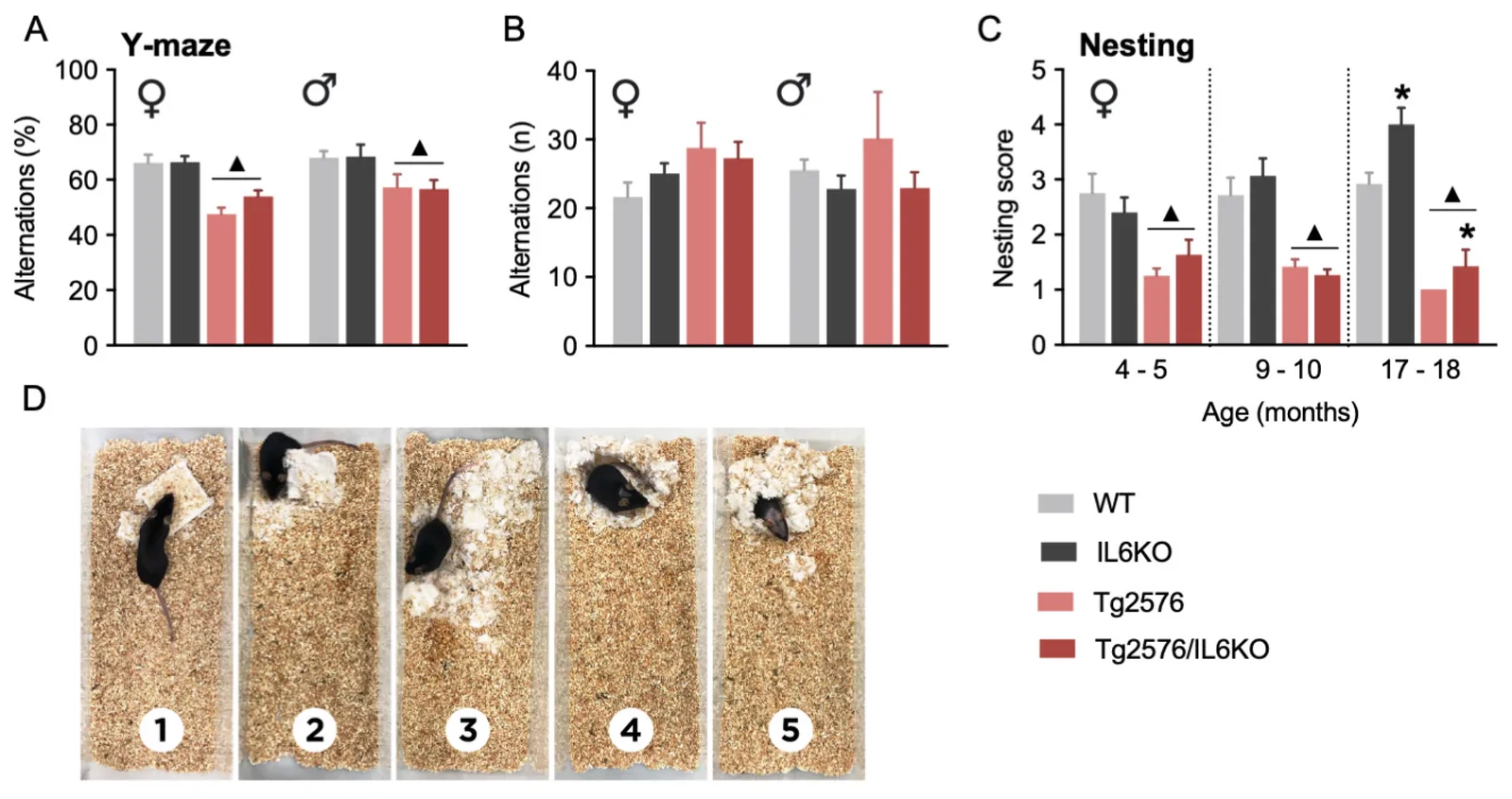
Effect of IL-6 deficiency on working memory and nest-building behavior in Tg2576 mice. (**A**) Impaired percentage of alternations in aged Tg2576 mice. (**B**) No differences in the total number of alternations. (**C**) Impaired nest-building behavior in Tg2576 females and effect of IL-6 deficiency in aged mice. (**D**) Representative images of the nesting score. Results are mean ± SEM; n = 11–16 in females, n = 10–16 in males (**A**,**B**); n = 5–16 (**C**). ▲ *p* ≤ 0.05 Tg2576 effect, * *p* ≤ 0.05 IL-6 deficiency effect.

A decreased percentage of spontaneous alternations in the Y-maze test revealed impaired working memory in Tg2576 mice (Figure 4A). Interestingly, this deficit was not associated with an increase in arm entries (Figure 4B). IL-6 deficiency did not appear to have a significant impact on working memory, although a trend towards improvement was observed in female Tg2576 with IL-6 deficiency.

### 3.4. IL-6 Modulates Nest-Building Behavior of Tg2576 Mice

We next assessed nest-building behavior, an innate hippocampal-dependent behavior that has been considered a measure of cognitive well-being. Tg2576 animals revealed impaired nest-building behavior, as indicated by a decrease in the average nesting scores. No effects were observed by IL-6 deficiency in Tg2576 animals. Interestingly, enhanced nest-building behavior was observed in IL-6-deficient mice, especially at aged stages (17–18 months old) (Figure 4C). Examples of low and high nesting score nests are shown in Figure 4D, highlighting the difference in overall nest quality.

### 3.5. IL-6-Deficient Mice Exhibit Decreased Aβ Peptide Levels in Serum and Increased Gliosis in the Brain

**Figure 5:**
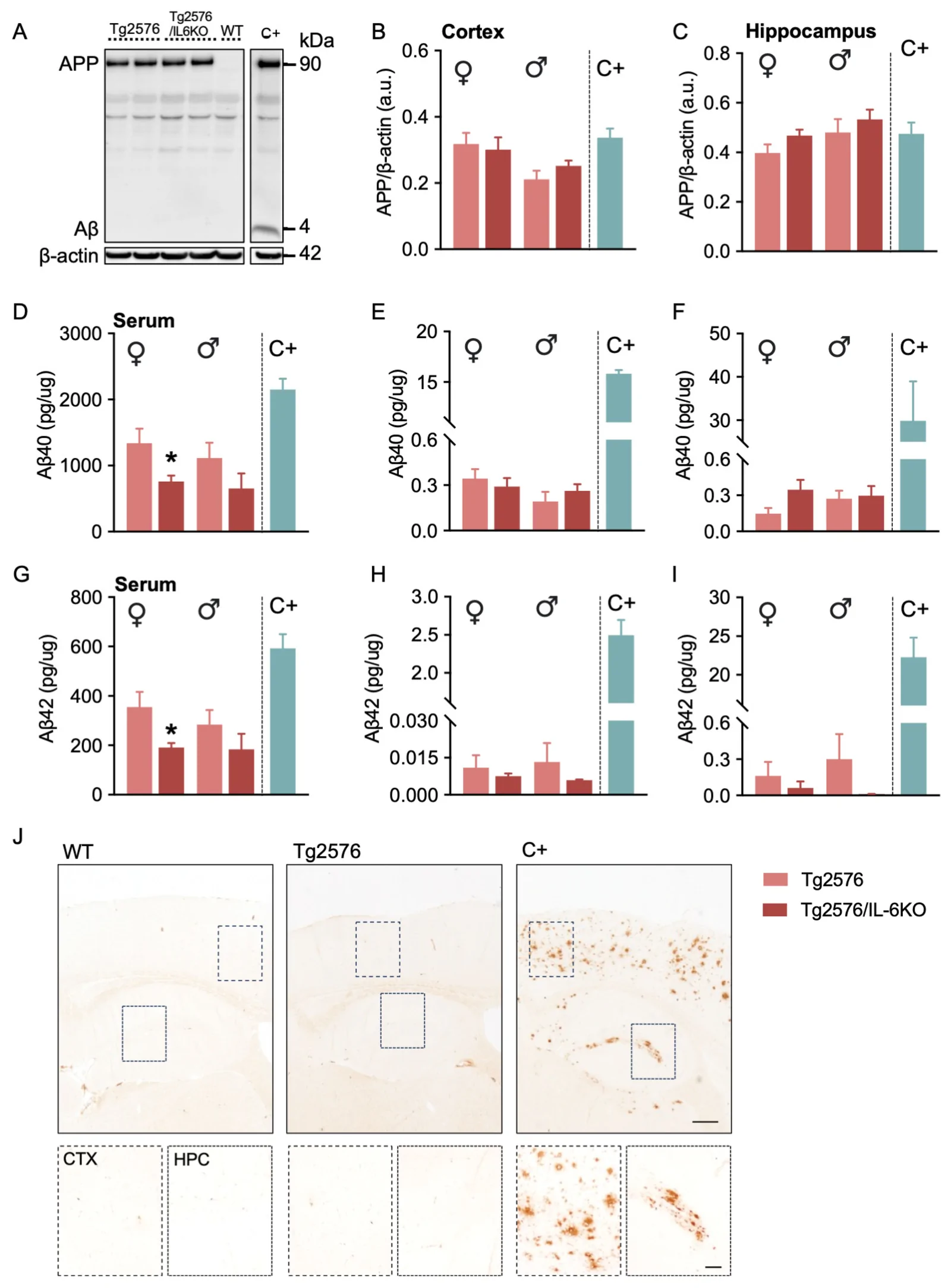
Effect of IL-6 deficiency on APP-/Aβ-related pathology in Tg2576 mice. (**A**) Representative immunoblot of APP detected with the 6E10 antibody; β-actin was used as the control protein. (**B**,**C**) APP levels in Tg2576 mice relative to β-actin in cortex (**B**) and hippocampus (**C**) are not affected by IL-6 deficiency. (**D**,**G**) Increased Aβ40 (**D**) and 42 (**G**) levels in serum samples of Tg2576 mice, partially reversed by IL-6 deficiency in females. (**E**,**F**,**H**,**I**) Absence of Aβ40 (**E**,**F**) and 42 (**H**,**I**) levels in cortical (**E**,**H**) and hippocampal (**F**,**I**) samples of Tg2576 mice, as compared to C+ samples. (**J**) Representative immunohistochemistry of 4G8 showing amyloid plaques in positive control samples; scale bar is 400 µm. Samples are compared to C+ samples, positive for Aβ levels in the brain (**A**–**J**). Results are mean ± SEM; n = 10–14 in females, n = 9–14 in males. For C+, n = 5–6 in WB, and 2–4 in ELISA. * *p* ≤ 0.05 IL-6 deficiency effect.

As expected, by Western blot Tg2576 mice showed elevated levels of human APP holoprotein in both cortex and hippocampus (Figure 5A), which was consistent with previously published data on these animals and confirmed by the positive control (C+). APP levels were quantified in both sexes of the different genotypes and were compared with a group of C+ mice (Figure 5B,C); they were similar in all groups both in the cortex and hippocampus, with no effect of IL-6 deficiency. Cortical and hippocampal levels of Aβ peptides were measured in 18-month-old animals using specific antibodies for Aβ_40_ and Aβ_42_ by ELISA. Unexpectedly, brain levels of these peptides were not detectable in cortical or hippocampal samples from Tg2576 mice, whereas both were readily detected in C+ samples (Figure 5E,F,H,I). The levels of Aβ in the serum of Tg2576 mice were measured. Tg2576 mice showed high levels of both Aβ_40_ and Aβ_42_, and there was a notable decrease caused by IL-6 deficiency (Figure 5D,G). The Aβ plaque load, as determined by IHQ, was also undetectable, as compared to C+ samples analyzed in parallel (Figure 5J).

**Figure 6:**
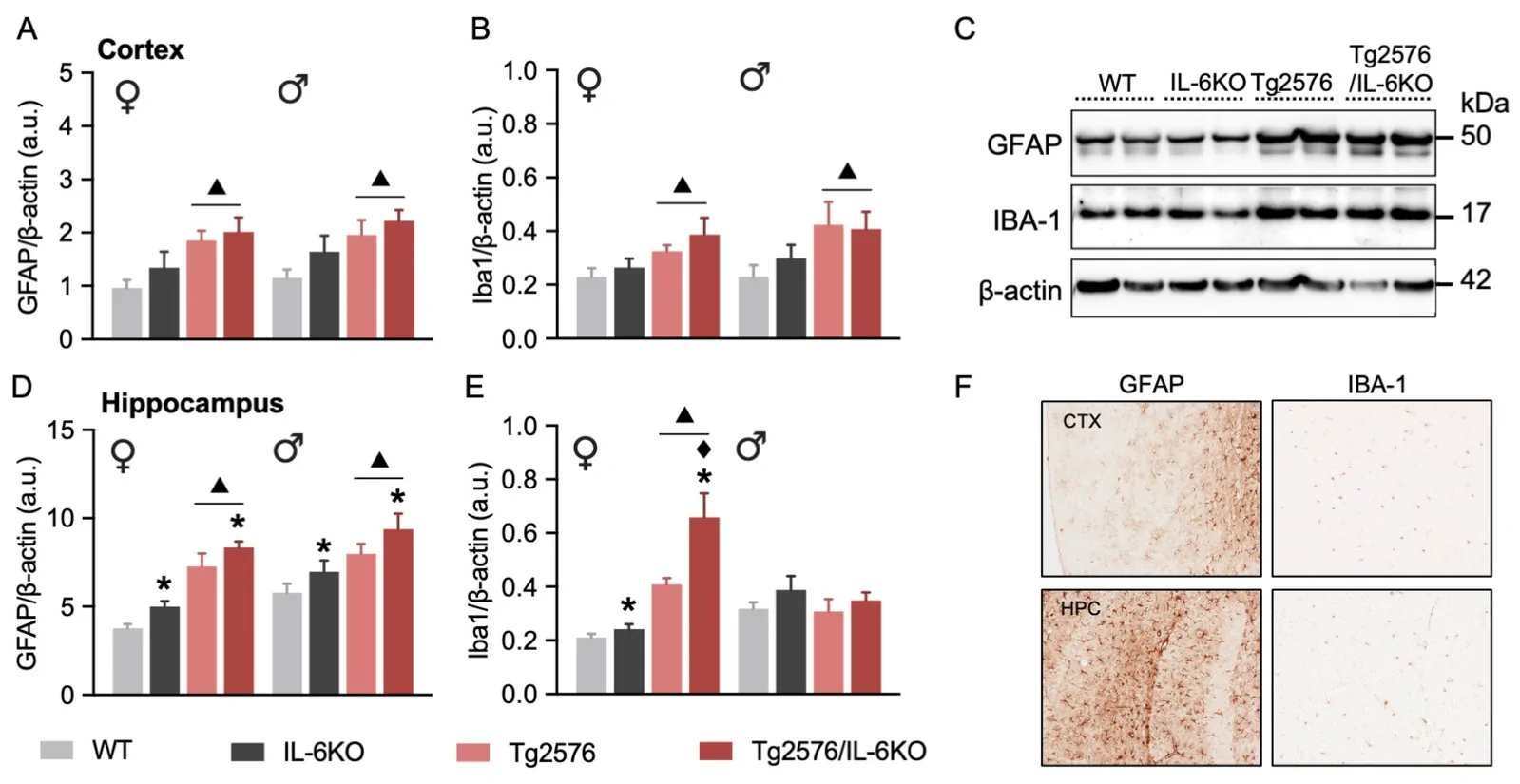
Effect of IL-6 deficiency on gliosis in Tg2576 mice. (**A**,**D**) Increased levels of GFAP and (**B**,**E**) Iba-1 levels in the (**A**,**B**) cortex and (**D**,**E**) hippocampus of Tg2576 mice and in IL-6-deficient mice, and its representative Western blots (**C**). (**F**) Representative immunohistochemistry of GFAP (left) and Iba-1 (right) of both cortex and hippocampus samples. Results are mean ± SEM; n = 9–10 females and males. ▲ *p* ≤ 0.05 APP negative, * *p* ≤ 0.05 IL-6KO, ♦︎ *p* ≤ 0.05 interaction between both factors.

Western blot analysis of total GFAP and Iba-1 in both cortex and hippocampus revealed an increase in both markers in Tg2576 mice (Figure 6A–E). Increased gliosis was observed in IL-6-deficient mice, especially in females. As expected by the lack of senile plaques, no increase in astrogliosis or microgliosis by IHC was observed in Tg2576 mice (Figure 6F, quantifications not shown).

## 4. Discussion

In the last decade, increasing evidence has highlighted a strong link between neuroinflammation and neurodegenerative diseases such as AD. Cytokines have been closely implicated in AD pathogenesis, with IL-6 emerging as a potentially important mediator [29,30,42]. In this study, we investigated the contribution of systemic IL-6 deficiency to the phenotype of Tg2576 mice, a well-established mouse model of AD that recapitulates several age-related physiological, behavioral, and cognitive alterations [11]. To this end, we crossed Tg2576 mice with a recently developed IL-6-deficient mice line [38]. Our findings indicate that IL-6 modulates several physiological, behavioral, and biochemical features of the Tg2576 phenotype. Importantly, our findings also show that these phenotypic alterations are not necessarily associated with established cerebral amyloidosis, highlighting both the complexity of the Tg2576 model and the context-dependent role of IL-6 in APP-related alterations.

Tg2576 mice exhibited premature mortality in both sexes, consistent with previous reports of reduced survival in this model. The mechanisms underlying Tg2576 mortality remain unclear. Several possibilities have been proposed, including altered synaptic plasticity and increased seizure susceptibility associated with APP or Aβ overexpression [43,44], developmental effects of APP and APLP (APP-like proteins) [45,46], cerebrovascular dysfunction, and oxidative stress [47,48,49]. Interestingly, systemic IL-6 deficiency partially rescued the increased mortality of female Tg2576 mice but not males. In contrast, IL-6 deficiency reduced survival in wild-type animals of both sexes. These findings further support the context-dependent nature of IL-6 signaling and suggest that its contribution to postnatal survival may depend on the underlying genetic background and sex. Previous studies have shown that IL-6 deficiency can increase susceptibility to different forms of physiological or pathological stress, including sepsis, LPS endotoxemia, and enterovirus lethality [50,51,52]. Studies in IL-6-deficient mice have also revealed increased brain damage and seizure susceptibility to various convulsant stimuli, suggesting that IL-6 is implicated in the modulation of basal synaptic transmission [53,54]. Seizures are also a typical manifestation in GFAP-IL6 mice, which overexpress IL-6 [55], while inhibition of IL-6 trans-signaling has also shown to increase survival [17]. Finally, previous studies on intrauterine life survival in mice with IL-6 deficiency revealed normal mendelian patterns [56]. However, a pro-survival role of astrocytic IL-6 was evident in a model of astrocytic IL-6 deficiency, indicating a possible cell-dependent role for IL-6 [57]. Thus, the opposing effects observed here may reflect differences in IL-6 source, signaling pathway, or interaction with APP-related processes. The mechanisms responsible for the marked sex difference in the response of Tg2576 mice to IL-6 deficiency remain to be established. However, these may be linked to genetic or epigenetic factors, or immune system differences [58].

Weight loss, a prominent early feature of AD [59], was also observed in Tg2576 mice compared to WT animals, together with reduced adiposity, and, particularly in females, tibia length, consistent with previous reports describing metabolic and skeletal alterations in this model [17,37,60,61,62,63,64,65]. IL-6 deficiency did not modify the decreased body weight or adiposity associated with the Tg2576 phenotype, regardless of sex. However, IL-6KO males, but not females, developed mature-onset obesity accompanied with increased adiposity. These findings are consistent with the metabolic syndrome observed in other studies performed in mice lacking IL-6, which show a progressive increase in body weight in males, regardless of differences in food intake [66], and increased body fat in aged IL-6KO males, along with altered glucose metabolism, dyslipidemia, and resistance to leptin [66,67,68,69]. IL-6, expressed in both adipose tissue and hypothalamic nuclei that regulate body composition, plays an important role in regulating body weight and body fat at the central level [67,69]. In fact, previous studies have shown that adipose-specific IL-6 deficiency regulates body weight and adiposity after high-fat diet in females but not in males, revealing strong sex-dependent effects of IL-6 in body weight [66]. At the central level, astrocyte-targeted overexpression of IL-6 resulted in resistance to high-fat diet in both body weight and body fat without altering food intake [70]. Mice overexpressing IL-6 also revealed reduced body weight and hyperinsulinemia [71]. Changes in the expression of the main hypothalamic neuropeptides involved in the regulation of energy homeostasis in both IL-6-deficient and -overexpressing mice suggest that IL-6 possibly regulates body weight via the expression of these factors in a sex- and age-dependent way [69,72].

The behavioral phenotype of Tg2576 mice was broadly consistent with previous studies, although the magnitude and nature of the alterations varied according to age and sex. Tg2576 mice displayed increased locomotor activity, enhanced exploration, and reduced anxiety-like behavior, particularly in females, in agreement with previous reports [15,16,17,37,49,62,73,74,75]. This has again been observed in our study in both young and aged mice, particularly in females. The anxiolytic-like behavior in Tg2576 has been explained in terms of disinhibitory tendencies [16,75], which are also reported in patients with AD [76], and that could be linked to a disruption of normal cholinergic function in these mice [77]. IL-6 deficiency consistently attenuated the increased locomotor activity observed in Tg2576 mice and, in aged animals, also reduced the increased exploratory behavior. These effects are consistent with previous evidence that IL-6 signaling contributes to the regulation of locomotor and exploratory behavior [17,34,35,36,37,39,57,78]. However, the effects on anxiety-like behavior were more complex and differed according to sex and age. IL-6 deficiency increased anxiety-like behavior in males, whereas aged Tg2576 females showed the opposite response. Such differences are consistent with previous observations in systemic, astrocytic, and microglial IL-6-deficient mice [34,35,39,78] and with the distinct behavioral effects produced by inhibition of IL-6 trans-signaling [17], thus highlighting the differences between trans-signaling and systemic IL-6 deficiency. These findings suggest that the behavioral consequences of IL-6 dysregulation cannot be considered uniformly pro- or anti-inflammatory, but instead depend on sex, age, cellular source, and signaling pathway.

Cognitive alterations were also strongly influenced by age and sex. Tg2576 males showed impaired spatial learning, particularly at older ages, together with impaired spatial reversal and retention, whereas females did not display a comparable cognitive deficit. These findings are broadly consistent with previous reports of age-associated cognitive impairment in Tg2576 mice, although substantial variability has been described between studies [11,14,15,17,37,79]. Remarkably, IL-6 deficiency improved spatial learning and memory predominantly in females. Because aged Tg2576 females did not exhibit a clear cognitive deficit relative to their controls, this effect may not represent a conventional rescue of an APP-dependent impairment, but rather a direct contribution of IL-6 to hippocampal function in females. IL-6 has been implicated in synaptic plasticity, learning, memory, and nervous system development [80,81], and some studies suggest that it might be negatively related to learning and memory formation, as blocking endogenous IL-6 in a “healthy” brain prolongs long-term potentiation (LTP) and improves long-term memory in hippocampal-dependent tasks [27,82]. The facilitatory effect of IL-6 deficiency on MWM performance was previously reported in young adult mice (4 months old) [83], but conflicting results were obtained in other studies that reported impaired learning in IL-6-deficient mice [36]. Surprisingly, the findings observed in the IL-6-deficient mice are contrary to what was observed after inhibition of IL-6 trans-signaling, which revealed mild memory impairment in the MWM [17], suggesting that different IL-6 signaling pathways play specific roles in the modulation of memory formation. Aged Tg2576 mice also showed impaired working memory, consistent with earlier findings of reduced spontaneous alternations in the Y- and T-maze, a measure that is sensitive to various brain lesions [73,74,84]. Finally, nesting behavior, an innate affiliative behavior crucial in mice for thermal regulation, reproduction, and shelter, has been demonstrated to be sensitive to hippocampal damage and the progression of neurodegenerative diseases [41,85]. Typically, changes in nesting behavior as failure to create a nest indicate a change in health or welfare. In line with our findings, poor nesting performance has been observed in AD mouse models, including Tg2576 and APP_SWE_/PS1 mice even from young ages, suggesting that other factors related to overexpression of mutant APP might be a contributing factor to this behavioral alteration [74,86,87,88,89,90]. However, IL-6 deficiency had no effect on the drastic decrease in nesting score in Tg2576 mice.

A particularly important finding of the present study was the absence of detectable prototypical cerebral amyloid pathology in Tg2576 mice at 18 months of age. Tg2576 mice are classically characterized by progressive secretion of Aβ_42_ and Aβ_40_ and to the formation of senile plaques with dense amyloid cores [11,49,91]. In our study, elevated APP levels were detected in brain tissues. However, neither Aβ_40_ nor Aβ_42_ was detectable in cortical or hippocampal samples, and amyloid plaques were not observed by immunohistochemistry. Importantly, well-established plaque-positive Tg2576 samples were included as positive controls and displayed clear cerebral Aβ pathology, in principle ruling out methodological problems underlying these unexpected results. The absence of detectable cerebral amyloidosis is therefore an important characteristic of the particular cohort analyzed here rather than an apparent consequence of the experimental approach. This finding warrants consideration of potential sources of variability in this model. As described, the animals analyzed here originated from Tg2576 founders purchased from Taconic at a different time from those used in previous experiments, whereas Tg2576-positive controls from other founders displayed the expected APP processing and cerebral amyloid pathology. These observations raise the possibility that colony- or cohort-related factors contributed to the discrepancy. Potential explanations include altered transgene dosage or expression, as transgene copy-number loss has been reported in other APP transgenic models [92], genetic modifiers that influence APP processing, Aβ production, or Aβ deposition, as genetic background can significantly modify brain Aβ levels in *APP*-transgenic mice despite comparable levels of unprocessed APP [93] or transgene silencing [94]. Environmental and husbandry-related factors cannot be entirely excluded, although experiments were conducted under the same experimental conditions and by the same experimenters as in previous studies [17,37], making these factors a less obvious explanation for the discrepancy. Although human *APP* expression was confirmed in the cortex and hippocampus, and total APP levels were comparable to those in plaque-positive controls and unaffected by IL-6 deficiency, transgene copy number, transcription, and APP-processing efficiency were not directly assessed. Thus, altered APP processing, reduced Aβ production, or increased Aβ degradation or clearance could explain the absence of detectable cerebral Aβ_40_ and Aβ_42_. Similar crosses have previously yielded the expected Tg2576 phenotype, making the IL-6-deficient genetic background a less likely explanation, although it cannot be completely excluded. Moreover, Aβ levels below the detection limits or the presence of other APP-derived fragments cannot be ruled out; our findings therefore indicate an absence of detectable cerebral Aβ_40_, Aβ_42_, and plaques rather than the complete absence of all Aβ species or APP-derived products.

Notably, circulating Aβ_40_ and Aβ_42_ remained elevated despite the absence of detectable cerebral amyloidosis, consistent with evidence that plasma Aβ does not necessarily reflect brain amyloid deposition [12]. Although *APP* is expressed in peripheral tissues, we did not detect APP processing in the heart or lung, and the origin of circulating Aβ therefore remains unresolved. Previous work has documented age-dependent changes in brain, cerebrospinal fluid, and plasma Aβ in Tg2576 mice [12]. Thus, peripheral production, altered peptide clearance, or differences in brain–periphery transport remain possible explanations, but cannot be distinguished with our data. These findings establish the absence of detectable cerebral Aβ and plaques in this cohort, rather than proving the complete absence of Aβ or APP-derived amyloidogenic products.

Despite this lack of amyloid deposition, these animals displayed several phenotypes previously described in Tg2576 mice, including altered survival, body weight, behavior, and cognition. This dissociation provides an opportunity to consider the contribution of APP-related mechanisms that do not require established amyloid plaque deposition. Previous studies have reported that behavioral and cognitive alterations can emerge in Tg2576 mice before substantial Aβ deposition [11,17,37,62,95]. Thus, recent research has shifted focus towards other mechanisms besides Aβ and its aggregated forms’ toxicity. The neurodevelopmental effects in Tg2576 mice could be explained by compensatory processes triggered by other genes in response to *APP* overexpression during brain development, to specific APP metabolites, and/or the background genes adjacent to it, which are inevitably inserted into the genome [95,96]. APP itself has important physiological functions in synapse formation, maturation, and maintenance, and altered APP signaling may therefore affect neuronal networks independently of extracellular Aβ accumulation [24,97,98,99]. In addition, APP C-terminal fragments, particularly C99, have been implicated in early APP-related pathological processes and cognitive dysfunction [18,19,20,21,22]. Cell type- and brain region-specific patterns of *hAPP* expression may also contribute to the phenotype of Tg2576 mice [23]. Thus, the phenotype observed in our animals could result from APP overexpression or altered APP processing rather than from the accumulation of amyloid plaques. Importantly, our data do not establish that the observed alterations are completely independent of Aβ, but they demonstrate that established cerebral amyloid plaque deposition is not required for the manifestation of several characteristic Tg2576 phenotypes.

Despite the absence of detectable Aβ_40_ and Aβ_42_ in cortex and hippocampus, Tg2576 mice displayed markedly elevated circulating levels of both peptides. Previous studies have shown that the *APP* transgene is expressed not only in the brain but also in peripheral tissues [12], providing a potential source of circulating Aβ. Consequently, although peripheral APP expression was confirmed, these analyses do not establish peripheral organs as the source of circulating Aβ. Moreover, studies in other APP-related models have demonstrated that increased plasma Aβ does not necessarily result in cerebral Aβ deposition [100]. Therefore, circulating Aβ levels should not necessarily be interpreted as a direct surrogate of cerebral amyloid burden. Interestingly, IL-6 deficiency reduced circulating Aβ_40_ and Aβ_42_ without altering total APP levels. This finding raises the possibility that IL-6 might influence APP processing or Aβ production rather than APP expression itself. However, because cerebral Aβ was undetectable in the present cohort, the functional significance of this reduction in circulating Aβ remains unclear. Differences in the genetic composition of the experimental and positive-control groups should also be considered when interpreting this comparison: the Tg2576 mice in the second experiment were heterozygous for the IL-6 allele following crossing, whereas the non-littermate positive controls were homozygous (see also below). This difference may have contributed to variation in circulating Aβ levels and limits the extent to which the groups can be directly compared. IL-6 has already been shown to interact with Aβ in multiple studies [26], as it has been found to be elevated in brains and CSF of AD patients [29,80], and brain administration of Aβ has been shown to be enough to elevate peripheral IL-6 levels in the periphery [101]. In fact, it has been suggested as a potential serum biomarker for AD [102]. Therefore, it is possible that various sources of Aβ may be responsible for this effect, including those in which IL-6 plays a role.

The absence of amyloid plaques was also accompanied by a relatively modest glial phenotype. Tg2576 mice showed increased total GFAP and Iba-1 protein levels in the brain, whereas immunohistochemistry did not reveal the pronounced astrocytic or microglial accumulation associated with plaque-containing brains. This pattern is compatible with the absence of established amyloid plaques, which are strong local stimuli for glial activation. Thus, while there was an overall tendency for them to be increased, this was only significant in the hippocampus. While clear, these increases were obviously lower compared to plaque-containing brains [37]. Interestingly, IL-6 deficiency further increased GFAP and Iba-1 levels, particularly in females. Given the well-established ability of IL-6 to regulate glial responses in mouse models [32,55,103], this increase may reflect altered glial homeostasis following the loss of IL-6 rather than a straightforward increase in pathological neuroinflammation. These findings further emphasize that IL-6 can exert complex and context-dependent effects on the brain.

In the crossing experiment discussed above, the WT and Tg2576 animals were heterozygous for IL-6, thus presumably with lower IL-6 levels. This was demonstrated in an experiment injecting LPS experiment in homozygous and heterozygous WT for IL-6, as expected (Supplementary Figure S1). Interestingly, that strongly suggests that Tg2576 mice appear to regulate IL-6 expression similarly to normal WT mice, at least in the context of the response to LPS. Whether or not the IL-6 haploinsufficiency would influence any of the results of the present study remains to be established.

## 5. Conclusions

Taken together, our findings provide evidence that the Tg2576 phenotype cannot necessarily be attributed to cerebral amyloid plaque deposition. Previous unsuccessful attempts to develop therapeutic approaches for AD targeting Aβ dysregulation (i.e., production or clearance) have already suggested that while Aβ may contribute to the pathogenesis of AD, it is not sufficient to cause cognitive decline or impairments in global functioning [104,105]. Tg2576 mice in the present study displayed multiple physiological, behavioral, and cognitive alterations despite the absence of detectable cortical or hippocampal Aβ_40_, Aβ_42_, and amyloid plaques. This observation is consistent with increasing evidence that APP and its proteolytic products can influence neuronal and synaptic function independently of established amyloid pathology [18,19,20,21,22,23,24,97,98,99]. At the same time, the marked increase in circulating Aβ demonstrates that APP-related amyloid production was not absent but rather dissociated from detectable cerebral deposition. IL-6 deficiency modified several of these phenotypes, reduced circulating Aβ, and altered glial markers, with effects that were strongly dependent on sex and age. These findings support a potential role for IL-6 in APP-related phenotypes and highlight the importance of distinguishing APP overexpression, Aβ production, and cerebral amyloid deposition when interpreting Tg2576 models of AD.

## Data Availability

The original contributions presented in this study are included in the article/Supplementary Material. Further inquiries can be directed to the corresponding authors.

## Supplementary Materials

The following supporting information can be downloaded at: https://www.mdpi.com/article/10.3390/cells15211921/s1, Figure S1: The IL-6 KO/WT line show haploinsufficiency compared to IL-6 WT/WT line.

## Abbreviations

The following abbreviations are used in this manuscript:

AD	Alzheimer’s Disease
IL-6	Interleukin-6
APP	Amyloid Precursor Protein
Aβ	Amyloid-β
PSEN	Presenilin
hAPP	Human APP
C+	Positive Control
OF	Open Field
HB	Hole Board
EPM	Elevated Plus Maze
MWM	Morris Water Maze
YM	Y-Maze
OAs	Open Arms
AUC	Area Under the Curve
PA	Platform Area
TQ	Target Quadrant
BAT	Brown Adipose Tissue
WAT	White Adipose Tissue
PFA	Paraformaldehyde
WB	Western Blot
ELISA	Enzyme-Linked ImmunoSorbent assay
BSA	Bovine Serum Albumin
DAB	Diaminobenzidine
GzLM	Generalized Linear Model
GEEs	Generalized Estimated Equations
APLPs	Amyloid Precursor-Like Proteins
