# Supplementary material for: Effects of Interleukin-6 Dysregulation in a Mouse Model of Alzheimer’s Disease: Unraveling Complexity Beyond Amyloidosis
Source: Cells. 2026 Oct 22;15(21):1921. doi: 10.3390/cells15211921 (PMC13761393; doi:10.3390/cells15211921)

To verify the haploinsufficiency of IL-6, an experiment injecting LPS was set up. IL-6 levels were dramatically increased by LPS in the serum, cortex and hippocampus in WT mice, to a lower extent in heterozygous WT mice, and undetectable in IL-6 KO mice. Tg2576 mice (heterozygous for IL-6) produced similar levels as heterozygous WT mice, whereas Tg2576/Il-6 KO mice showed undetectable IL-6 levels, as expected.

- Drugs and injections. Lipopolysaccharide (LPS) from Escherichia coli (O55:B5, Sigma-Aldrich, St. Louis, USA) dissolved in physiological saline (0.9% NaCl) was injected intraperitoneally at a concentration of 0.5 mg/kg in a volume of 0.1 ml / 10 g animal, or the same volume of saline. Mice were anesthetized intraperitoneally with a ketamine-xylazine (K, 100 mg/kg; X, 16 mg/kg) solution 4 h after the LPS injection. Blood was collected by cardiac puncture with a heparinized syringe. Then, animals were transcardially perfused with physiological saline before brains were extracted, and hippocampus and cortex were sectioned and immediately frozen in liquid nitrogen. Blood was centrifuged at 6000 x g for 10 min at 4 ºC to separate the resulting supernatant as plasma.
- Luminex multiplex assay. Luminex multiplex assay was performed to measure the abundance of IL-6 using the MILLIPLEX MAP Mouse High Sensitivity T Cell Magnetic Bead Panel (Merck Millipore MHSTCMAG-70K, Merck, Darmstadt, Germany). Cortex and hippocampus samples were used at a protein concentration of 1-7 µg/µl of the tissue lysate, diluted in protein extraction buffer if needed, and serum samples were diluted in 1:12 in Serum Matrix provided by the kit. The protocol was performed according to the manufacturer's recommendations. The resulting data were acquired using a validated and calibrated Luminex MAGPIX® Instrument System (powered by Luminex xMAP technology, Luminex Corporation, Austin, USA) and analyzed using the xPONENT 4.2 software (MAGPIXXPON42, Luminex Corporation, Austin, USA). The lysate protein concentration was used to normalize the data. Results obtained below the first standard of the curve were considered as 0, and some cytokines levels were unquantifiable and thus were not plotted.

Supplementary Figure S1. The IL-6 KO/WT line show haploinsufficiency compared to IL-6 WT/WT line. *In vivo* response to LPS. (A) IL-6 protein levels in plasma after Vehicle or LPS injection. IL-6 protein levels in cortex (B) and hippocampus (C) after Vehicle or LPS injection. Results are mean ± SEM and analyzed by GzLM statistical test; ï $p \leq .05$ vs vehicle, ▲ $p \leq .05$ vs WT(het), Δ $p \leq .05$ vs Tg2576.

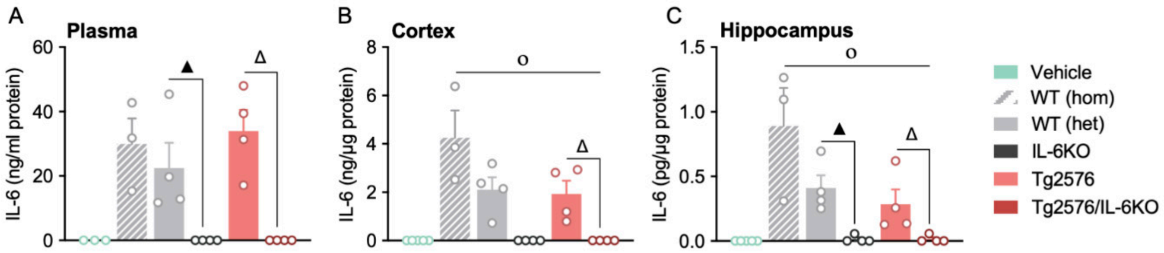

Supplement: Supplementary file 1 [file cells-15-01921-s001.zip › cells-4580764-supplementary.pdf]
